# Software selection in large-scale software engineering: A model and criteria based on interactive rapid reviews

**DOI:** 10.1007/s10664-023-10288-w

**Published:** 2023-02-28

**Authors:** Elizabeth Bjarnason, Patrik Åberg, Nauman bin Ali

**Affiliations:** 1 Department of Computer Science, Lund University, Lund, Sweden; 2 Ericsson AB, Lund, Sweden; 3 Department of Software Engineering, Blekinge Institute of Technology, Karlskrona, Sweden

**Keywords:** CBSE, Component-based, Component selection, Tool selection, Rapid review

## Abstract

**Context:**

Software selection in large-scale software development continues to be ad hoc and ill-structured. Previous proposals for software component selection tend to be technology-specific and/or do not consider business or ecosystem concerns.

**Objective:**

Our main aim is to develop an industrially relevant technology-agnostic method that can support practitioners in making informed decisions when selecting software components for use in tools or in products based on a holistic perspective of the overall environment.

**Method:**

We used method engineering to iteratively develop a software selection method for Ericsson AB based on a combination of published research and practitioner insights. We used interactive rapid reviews to systematically identify and analyse scientific literature and to support close cooperation and co-design with practitioners from Ericsson. The model has been validated through a focus group and by practical use at the case company.

**Results:**

The model consists of a high-level selection process and a wide range of criteria for assessing and for evaluating software to include in business products and tools.

**Conclusions:**

We have developed an industrially relevant model for component selection through active engagement from a company. Co-designing the model based on previous knowledge demonstrates a viable approach to industry-academia collaboration and provides a practical solution that can support practitioners in making informed decisions based on a holistic analysis of business, organisation and technical factors.

## Introduction

Today’s software products and development environments consist of a wide range of software components and tools used in software-based products, and in manual, as well as, automated software development toolchains for performing tasks such as automatic tests and continuous integration and deployment. Software from various sources, such as commercially available components and systems, open source software communities, is selected and combined with software developed in-house to provide the required behaviour and support. As the knowledge and amount of methods, techniques, and tools for software development have increased over the year, so has the challenge of designing and selecting software components for a software development organisation (Du Plessis 1993).

While there exist numerous models for selecting software, research indicates that practitioners mostly still use impromptu approaches to select software (Ayala et al. 2018). In a case survey, Petersen et al. (2018) investigated the state of practice regarding component sourcing decisions and concluded that “decision-making approaches” used in practice are “mostly ad-hoc and not well structured”. Furthermore, Petersen et al. found that existing recommendations in the literature on criteria to consider in the selection process are often not followed in practice (Petersen et al. 2018). Thus, there is a need for practically applicable guidelines that take a holistic approach to decision making w.r.t. software components.

The weak uptake in industry may be due to that many of the previous models were developed without extensive collaboration with industry, and thus lack industrial relevance. Research on the transfer of technology and industry-academia collaboration has shown that high levels of cooperation is a success factor in achieving industry adoption of research findings (Wohlin 2013; Garousi et al. 2016; Engström et al. 2017).

Since the aim of our work is to provide a practically usable model that can support software development organisations in selecting and evaluating software components and tools, we performed this research in close collaboration with a case company. We had the explicit ambition to work as “one team” with a high degree of engagement from industry. This was achieved by applying method engineering to an industry-specific problem, and by jointly designing a solution to this problem. The co-design was achieved through an iterative and gradual process with frequent communication and interaction between industry and academia. This close collaboration and mutual engagement were further facilitated by using the systematic approach of interactive rapid reviews (Cartaxo et al. 2020; Rico et al. 2020). Findings from previous research were thus identified and used as input to the design of our software selection model, together with practitioner insights. The model has been validated through a focus group and through re-analysis of the literature identified in our rapid reviews. In addition, we provide an example of use where our model was applied at the case company to select software for a tool extension.

Our model supports a holistic perspective that considers the overall business, organisational, and technical environment in which the software is to be used. This contrasts previous research that mainly focuses on criteria for functional aspects, e.g. for CASE tools (Kornecki and Zalewski 2005). Thus, our model also includes organisational and business aspects of the overall environment and related ecosystem, in addition to the functional requirements for the specific components that are to be selected. The selecting organisation is responsible for identifying the requirements and priorities that are specific to their needs and goals, thereby providing a flexible model. This in-built flexibility enables practitioners to consider and optimise the cost-benefit ratio of the selection process, by focusing on the criteria most relevant to their current needs.

The remainder of the paper is structured as follows: Section 2 presents the related work. Section 3 describes our research method. In Section 4, we present the software selection model, and in Section 5, we report the results from validating the model through a focus group at Ericsson. Finally, we discuss threats to validity and limitations of our study in Section 7 and present our conclusions in Section 8.

## Background and Related Work

The decision making processes around selecting software have been researched for different contexts of use, in particular for software tools and products released to customers and end-users. While the context of use affects some criteria and priorities of these, there are also overlaps and similarities between these two contexts. For this reason, we based our study on a combination of literature on software selection for software engineering tools and for component selection for production software. This includes literature on computer-aided software engineering (CASE), selecting tools to support continuous software engineering, using quality criteria to select software components, and component-based software engineering (CBSE).

We have used the studies described here in our design to obtain comprehensive coverage of known considerations for selection decisions. There is a lack of consensus and varying prioritization of influencing attributes in the literature. This indicates a need for further research on this topic and for a selection model that allows practitioners to choose the essential factors in the specific context of their selection decision. Furthermore, with our collaborative approach (working as a team with practitioners) to co-produce the software selection model, we attempted to overcome the limitation of previous models where researchers have (at best) only had passive input from the industry.

### Computer-Aided Software Engineering (CASE)

Supporting the tasks of the development process with computer-aided tools (CASE tools) has been considered since the 1960s when software engineering became a discipline. We find that the majority of research on criteria for software selection in the CASE context took place during the 1990s, and an international standard was published in 1995. The ISO/IEC 14102:1995 contains guidelines for evaluating and selecting CASE tools, and was updated about a decade later and replaced by the ISO/IEC 14102:2008. The standards provide a systematic method (process) for evaluating and selecting CASE tools based on organisational requirements, a set of CASE-tool characteristics, and measurements for these characteristics. Lundell and Lings reviewed the literature on CASE tool selection, and commented that the 1995 standard was neither complete nor consistent, and that the mainly quantitative approach of the standard needs to be complemented by a qualitative approach to take into account the subjectiveness of human assessment (Lundell and Lings 2002).

The ISO/IEC 20741:2017 provides a generic four-step process for the evaluation and selection of any software engineering tools, i.e. not only CASE tools, consisting of preparation, structuring, evaluation, and selection. This process maps well to the one proposed in our selection model. This standard also contains a set of generic criteria for performance, compatibility, usability, reliability, security, maintainability, and portability. For tool-specific characteristics, the ISO/IEC 20741 refers to tool-specific standards. Our model takes this one step further and also considers software components that are used as part of production software, thus broadening the scope of applicability of the selection process beyond that of tools.

The research on selecting and evaluating CASE tools consider several different topics, including how to evaluate tools (Du Plessis 1993; Lundell and Lings 2002), relevant requirements, factors, and criteria for tools in general (Lending and Chervany 2002; Biffl et al. 2009) and for specific development tasks, e.g. configuration management (Bashroush et al. 2017), mutation testing (Delahaye and Du Bousquet 2015), and domains, e.g. military defense (Alnafjan et al. 2013), small-and-medium-sized enterprises (Rivas et al. 2008). While the research on task-specific CASE tools mostly provides input into functional and quality requirements for these specific tools and domains, this research also provides insights into aspects and factors that are relevant to the generic topic of software selection. For example, the systematic literature review on CASE tools for variability management by Bashroush et al. identified several generic aspects as important, such as usability, alignment with current development practices and tools, standards adherence and quality requirements regarding resource constraints (Bashroush et al. 2017). Similarly, the work by Alnafjan et al. on selecting CASE tools for military applications identifies generically relevant criteria, such as vendor state, cost, and security (Alnafjan et al. 2013).

Delahaye and Du Bousquet observed that criteria and requirements on usage scenarios are of less value when there are only a few available tools, or when there is a single overwhelming factor (such as cost) that dictates the selection decision. In other cases, when there exists a set of suitable tools, they recommend using a systematic assessment approach based on criteria, but also to focus the evaluation effort based on the intended usage scenarios (Delahaye and Du Bousquet 2015).

Lending and Chervany observed that research on CASE tools provides conflicting accounts of the impact these tools have on productivity and software quality, and performed a multi-case study to investigate possible causes of these varying degrees of tool success. They found that developers are less enthusiastic about using restrictive tools since they find them less useful, and suggest adopting a broad perspective when selecting tools, beyond focusing solely on functionality and cost (Lending and Chervany 2002). When selecting and introducing a new software tool, the users’ perceptions and satisfaction thus play an important role in maximising their intentions to use it.

### Tool Selection for Continuous SE

Numerous software tools are available and used today in the toolchain that supports continuous software engineering (Shahin et al. 2017; Li et al. 2018). Kersten (2018) describes the number of tools available and used in modern software development as a Cambrian explosion of tools. They emphasize the need for an end-to-end perspective. This end-to-end view is operationalized by Jabbari et al. (2018), who present the use of a benefits dependency network to identify what process changes and tools are required to achieve the business goals. To define an approach to reach this state, they suggest identifying the necessary practices and the required order of implementation. As the last step, the technology required to facilitate implementation is identified. This framework provides an approach for aligning technology implementation with business objectives.

Modern software development has been influenced by cloud-based software infrastructure. In particular, for deploying and managing the applications in the cloud. In a systematic mapping study, Pahl et al. (2019) identified the motivation for using containerized systems, their development and management, and the technologies used. They identified several facets of quality that can be used as parameters for selecting a tool/technology. Three broad categories of quality parameters include (1) monitorable parameters (like performance, resource utilization, startup time, security, reliability, workload, size/volume, and compliance), (2) non-monitorable parameters (like portability and interoperability), and (3) system parameters (like scalability and reconfigurability).

### Quality Assessment Criteria for Software

Several criteria have been proposed to evaluate the quality of software components both in the closed-source and open-source context. Adewumi et al. (2016) identified 19 quality assessment models for open-source software. Similarly, Goulão and Abreu (2004) identified nine models for evaluating a component or a collection of components. One main difference between the proposals for the two contexts is considering community-related factors in OSS models, e.g., developer activity, market success, and sustainable community. We used these aspects to derive the models.

### Component Based Software Engineering (CBSE)

Component-based software engineering is a development approach where existing components are used to meet user requirements, instead of developing software from scratch. When choosing a component, practitioners consider different aspects and base their selection decisions upon these, often based on expert judgement (Borg et al. 2019). The search and evaluation of candidate components are often performed in an ad hoc fashion without any documented process and the decisions are based on the practitioner’s own experience (Ayala et al. 2018).

There are several research studies on which aspects that are considered in the industry when selecting components. Aspects commonly used by practitioners include the cost of acquisition, integration and licensing, sourcing options, and the quality of the component. In a systematic literature review, Badampudi et al. found that component decisions mainly focus on in-house vs COTS (Commercial Off The Shelf), and COTS vs. OSS (Open Source Software), and identified factors affecting these decisions, such as cost and reliability (Badampudi et al. 2016). However, research on the exact set of aspects to consider when selecting software is inconclusive. We pose that these variations in research results may be due to contextual differences, thus indicating a need for a selection model that can be adapted to the specific situation and context at hand; a perspective that has been included in our selection model.

Several surveys have been performed of which aspects practitioners use and prioritise when making decisions on component selection. For example, Chatzipetrou et al. (2020) asked practitioners to prioritize 12 categories of attributes that may influence their component selection decision and received 157 responses. They grouped several important factors like licensing and vendor relations in the “others” category. Chatzipetrou et al. concluded that cost (development, licensing, and maintenance cost) is the primary consideration in component selection decisions. Three other main attribute categories were support for the component, evolution of the components, and their functional fitness. In contrast, a survey performed by Borg et al. indicated that functional suitability was the most prioritised aspect for make-or-buy decisions in CBSE, followed by the reliability of the components (Borg et al. 2019). Finally, Carvallo et al. (2007) suggest using ISO-9126 software product quality standard as a base catalog for attributes in component selection criteria and also suggest organising selection criteria into higher-level categories consisting of lower level, observable and measurable properties.

Researchers have also proposed decision-making models that consider various ranking criteria for selecting components (Garg 2022). However, this study focuses on decision-support models that support discussion and argumentation in component selection.

Several component-selection models have been proposed (Wohlin et al. 2016; Alégroth et al. 2019; Nazir et al. 2014) and Alégroth et al. (2019) pose that any component selection model can be assessed based on seven characteristics. These characteristics are understandability, time taken to arrive at a decision, adaptability to different contexts, flexibility in application, decision support for the practitioners, the accuracy of the model and quick feedback on decisions. Alegroth et al. put forward a proposal for component selection, compared it to a selection process proposed by Wohlin et al. (2016), and found that their proposal fared better on several of the dimensions listed above.

Wohlin et al. (2021) presented a roadmap for future research in component selection. Reviewing the models listed above, they suggest investigating factors that can help understand the context for the decisions regarding component selection to choose among simple or more complex decision models. However, their main conclusion regarding further research in this area is the following: *“Regardless of approach, any future decision modeling requires a strong industrial base to stand on, which implies that the research should be conducted in close collaboration with industry and focus on incremental improvements of state of practice rather than introducing a new set of practices. Although the latter may be more impactful, the former is more likely, from our experience, to succeed.”*

We followed the above recommendation in this study. We collaborated closely with industry and leveraged existing decision-making models.

## Research Method

**Fig. 1:**
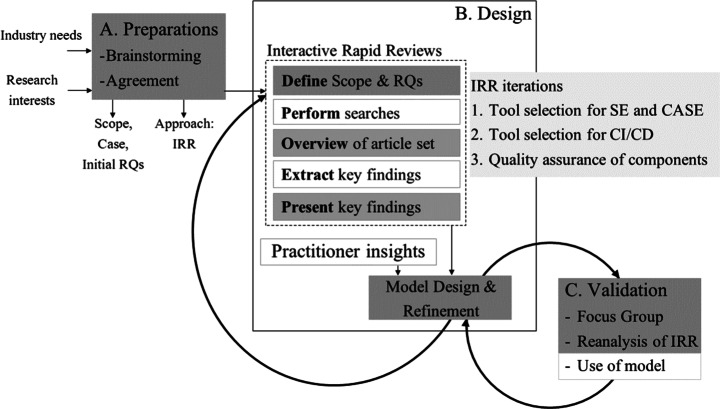
An overview of our research method. Three interactive rapid reviews (IRR, iterations 1-3) provided input to our iterative design process together with practitioner insights. Activities marked in dark grey were performed by academics and practitioners in collaboration

Our study was performed in close collaboration with Ericsson AB and consisted of three main stages, namely (A) *Preparations* (to identify scope and approach of study), (B) *Design* (of software selection model), and (C) *Validation*. In the design stage, we applied an iterative approach with several increments where rapid reviews were used as input to the design together with industry insights. An overview of our research method is shown in Fig. 1, where joint activities are highlighted in dark grey.

Our research approach was inspired by the systematic approach of method engineering (Brinkkemper 1996) where new methods are derived iteratively through searching for existing solutions, adapting and testing these. Furthermore, we used interactive rapid reviews (IRR) (Cartaxo et al. 2020; Rico et al. 2020) for performing systematic literature reviews in a way that supports close interaction with practitioners and thus combines a scientific approach with close collaboration with industry. The rapidness is achieved by compromising the rigour of a literature review, e.g., reducing the extensiveness of the search and whether or not multiple reviewers are including and excluding papers. The approach still follows a procedure and documents the “shortcuts”/“decisions” to prioritize quick feedback over an unbiased systematic investigation. Throughout a rapid review, researchers present and discuss goals and intermediate findings with practitioners, and together shape goals and research questions thereby enhancing the relevance of the research. Rapid reviews also provide immediate value to practitioners by presenting evidence-based insights throughout the study.

Using RapidReview for the literature review guided us to consciously discuss, decide and communicate the roles and expectations of the practitioners and the researchers participating in the study. The proposed iterative process of a rapid review was also helpful as the literature search was triggered by the new information needs that arose as the collaboration progressed.

Our case company, Ericsson AB, is a leading provider of Information and Communication Technology including technology for 5G mobile networks and IoT platforms. This study has been performed with the company’s software technology research programme through collaboration with the 2nd author and with people from one of the company’s units responsible for software engineering tools for their base-band infrastructure development.

A distinctive feature of our study is the extent of interaction and collaboration with the industrial partners in all stages of the research, from defining the scope and research method through the collaborative design of the selection model to the validation of it in the focus group. In total, this collaboration included fourteen pre-booked meetings with the company representative and one focus group meeting. In between these meetings, the authors communicated via e-mail and by sharing intermediate work products. Furthermore, both the involved researchers and the practitioners had additional meetings with “their own side” to discuss and synchronise the work. For example, the second author discussed intermediate versions of our selection model with others within the case company, thereby further improving the industrial relevance of the model. In addition, the second author independently used the model in another research project to select software required in that (other) project.

### Stage A: Preparations and Goal Setting

In the initial stage of our study, the industry and academia parties discussed and agreed to the initial *scope* and overall *approach* of our joint research, and a set of initial *research questions* were defined. As prescribed by the guidelines for rapid reviews (Rico et al. 2020), this included discussing the goals and aims of the study through a set of meetings involving the first and the second author. For both authors, the overarching aim was to encourage industry-academia collaboration within software engineering by providing an example of how and what value such collaboration can bring to industry. Through sharing and discussing current industry needs and research interests of the involved parties, an initial concrete goal was defined as investigating how to assess and select software tools. This goal was selected since this posed an unsolved and relevant problem at the company, and was one for which there exists previous research findings. The initial research questions, which were later revised as the work progressed, were defined as:

- What criteria are relevant for Ericsson to consider when selecting a SE tool?
- How can cost and benefit be considered and balanced when selecting SE tool solutions?
- How is the selection of an SE tool affected w.r.t. the aim to improve a) overall productivity and b) product quality?

We selected interactive rapid reviews as the overall *approach* for the work. This approach was selected since it allowed us to draw from the existing knowledge base in a (relatively) efficient manner, which facilitates industry-academia collaboration. Choosing to apply rapid reviews in this study, also provided a case for evaluating the work on rapid reviews within software engineering performed by colleagues within the research group, in which the third author is involved.

As the work progressed in the design stage (stage B), the topic under investigation was extended from software tools to software components in general, since both concern the assessment and selection of software, and thus have a lot in common. The scope was also extended from merely identifying criteria to also describing a high-level process for the selection of software components. These changes in scope were driven by our industry partner, and agreed to by all authors.

### Stage B: Iterative Design based on Rapid Reviews

The model presented in this article was designed through three main iterations, each consisting of a rapid review of current research. The articles and key findings from literature were presented to and discussed with our industry partner. This then provided input to the model design. The rapid reviews performed for each iteration are outlined in Table 1 and described below. For each iteration, the scope and research questions for the specific rapid review were *defined* in collaboration with the industry partner, and the academic partner then *performed searches* for relevant literature. To handshake and validate the relevance of the identified set of articles, an *overview* of the set was presented and discussed between industry and academic partners. The papers in the set were then reviewed by one of the participating researchers (author one for the first iterations, and author three for the remaining iterations) and the key findings were *extracted* and coded. These findings were then *presented* to the other authors and discussed, and used as input to the design of the model together with *insights into current practice* provided by the second author based on his industrial experience.

**Table 1:** Overview of the rapid reviews providing input to each design iteration

It.	Scope	No of hits	Full set	After validation
1	Tool selection for SE/CASE	147 + 20	31	25
2	Tool selection for CI/CD	113	14	6
3	Quality assessment of candidate components	237	36	6

In the first iteration, searches yielded a number of hits, which were reduced after applying inclusion/exclusion criteria resulting in a **full set** of articles which was then analysed. This set was reduced somewhat **after validation** of the full set. For iterations 2 and 3, the focus was on identifying existing secondary studies

In the first design iteration (1), which focused on selection criteria (for software tools), two separate lists of criteria were used as input to the design (available online, see Bjarnason (2021)). One of these lists was derived by the first author based on the rapid review of evaluation criteria for software tools. The other list of criteria was produced by the second author based on his long and extensive industrial experience with such tools. Together, these two authors designed the first version of the software selection model by reviewing, comparing, and discussing the criteria from each list through a series of face-to-face meetings.

In later design iterations, this initial version of the model was gradually extended to also contain a high-level process for identifying, assessing, and screening solution options. This process was inspired partly by ISO/IEC 20741:2017 and designed to fit our case company. The subsequent design iterations were supported by two additional rapid reviews (2 and 3) on the related topics of software component selection for continuous integration & deployment toolchains and quality assurance of software components, and by industrial insights provided by the second author. The additional rapid reviews were performed by the third author, who was invited to join the study and to contribute with additional insights into software selection.

#### Iteration 1: Criteria for Selecting Tools

For this first iteration of the design stage, the initial version of the research questions of the overall study were used as the search objective for this rapid review, namely criteria for selecting software tools.

The search and selection of literature for this iteration of the rapid review was performed in Scopus by the first author. The main objective of this rapid review was to provide a starting point for the design, rather than performing an exhaustive search of all relevant literature. Thus, a narrow search string was selected. After exploring the topic through different test searches, the following search string was defined: ALL (select* ) AND TITLE (tool* ) AND TITLE-ABS-KEY (“software engineering tool” ) OR TITLE-ABS-KEY (“CASE tool” ). Only peer reviewed articles written in English were included.

The initial search yielded 147 hits, which were then gradually scanned (title, abstract, and full paper). The following type of articles were included:

1. present or evaluate criteria for assessment and/or selection of tools for industrial software engineering work
2. discuss selection, assessment or evaluation of SE tools (including CASE tool)
3. empirical investigations of experience of introducing or using a tool.
4. articles that are borderline or when unsure of the relevance of article content, until later screening steps.

The following type of articles were excluded:

1. articles that describe the design and/or implementation of specific tools mainly from a feature perspective, including investigations into detailing functionality for specific domains or applications.
2. articles that solely evaluate the impact of tools on productivity etc without investigating explanatory factors
3. articles on non-software tools and/or tools in a non-industrial software engineering context
4. publications that are not scientific articles, e.g. presentation slides
5. articles that have later been extended (include the extended version)

For all articles included after the title scan, key information about the title, authors, publication forum, year, and abstract were extracted and placed in a spreadsheet (available on-line (Bjarnason 2021)) and used for the subsequent selection steps (abstract and full article). Notes were made on reasons for inclusion and exclusion, and an initial categorisation of the articles was performed. This resulted in a final set of 27 articles to which we added 4 additional articles from complementary searches performed by the third author, who was invited to join the study at this point. The third author searched for literature on component selection, to broaden the search space beyond that of tool selection. These complementary searches yielded 20 articles, of which four were especially relevant and selected to be included in the first iteration. Thus, the full set of articles for the iteration consisted of 31 articles. An overview of this full set was compiled and presented to our industrial partner, i.e. the second author. This presentation material is available on-line (Bjarnason 2021). The main purpose of this meeting was to validate the relevance of the found set before proceeding with extracting key findings.

The pdfs for the articles in the final set were imported into nVivo for analysis to identify the key findings. Through thematic coding of the articles, a number of selection criteria and categories of these were identified such as *functionality*, *usability*, *artefact quality*, *user support*, *productivity*, and *tool quality*. In parallel, the involved practitioner (second author) made a list of selection criteria based on his industrial experience and through talking to colleagues within the case company. Both lists are available online, see Bjarnason (2021).

The two lists of criteria, one from the rapid review and one based on practitioner experience, provided the starting point for designing the first version of our selection model. The first and second author meet (in person) to present and discuss the items on both lists. The outcome of these meetings was a merged set of selection criteria. This was the first version of our selection model, which was later extended and revised.

#### Iteration 2: Tool Selection for CI and CD

While there are differences in selecting software that will become part of the end product and software used to facilitate the end product’s development, deployment, and operations, we decided that the model should be generic enough to handle both contexts. To identify research on tool selection in the modern context, we used the following search string in Scopus: (“systematic review” OR “systematic literature review” OR “systematic mapping” OR “systematic map”) AND (“infrastructure as code” OR “continuous deployment” OR “continuous integration” OR “continuous delivery” OR “DevOps”) AND (“tool” AND “select”). This search string resulted in only four hits in Scopus. We conducted another search in Google Scholar that gave 113 hits using the search string [allintitle:(DevOps OR “continuous integration” OR “continuous delivery” OR “continuous deployment”) tools]. In this iteration, we included a paper for full-text reading if it met the following two conditions:

- the paper is in the particular context of continuous software engineering (Bosch 2014)
- the paper presents a survey, review or overview of solutions, challenges or any other related aspect of tool selection.

After screening the titles and abstracts, eight articles were selected for full text reading. The search results included the following systematic secondary studies and surveys (Shahin et al. 2017; Li et al. 2018; Kersten 2018; Jabbari et al. 2018; Pahl et al. 2019; Wurster et al. 2019).

These studies demonstrate the challenge of selecting software tools in modern software development, which further motivates the need for the model and the criteria developed in this study.

#### Iteration 3: Quality Assessment of Candidate Components

The quality of candidate software was one of the central dimensions in the existing component selection models and the emerging design in our study. Therefore, we decided to search for quality models for assessing software explicitly in Scopus. TITLE-ABS-KEY ((“systematic review” OR “systematic literature review” OR “systematic mapping” OR “systematic map” OR survey ) AND (“software component” OR “open source” ) AND (“quality” AND (“assessment” OR “model” OR “metrics” ) ) ) AND (LIMIT-TO (SUBJAREA , “COMP” ) )

In this iteration, we used the following selection criteria. For a paper to be included for a full-text reading, it should:

- propose, collect or review metrics, criteria, processes or models for assessing software component or system quality.

36 items were read in full-text and the following reviews (Adewumi et al. 2016; Miguel et al. 2014; Goulão and Abreu 2004; Abdellatief et al. 2013) and models (Sen et al. 2012; Alvaro et al. 2007) were identified.

The quality criteria identified in these studies were presented to the practitioner and were considered for inclusion in the proposed model.

### Stage C: Validation

The model, resulting from the rapid review iterations, was validated at the case company through a focus group and by applying the model to a practical example. In addition, a re-analysis of the previously identified literature was performed to locate evidence for each part of the model and to search for potentially missed criteria or aspects. The outcome of this re-analysis is provided in the description of our model through references to previous work identifying these aspects and criteria.

The focus group was held with four experienced practitioners, including the second author of this paper, to present, discuss, and validate our software selection model. The other three participants in the focus group were selected from a unit within the company that is responsible for internal software development tools, see Table 2.

**Table 2:** Focus group participants

Role	Years in software development
Manager of tools team	20+
Product owner of HW-based tool solution	20+
Product owner of testing tool	5

At the meeting, we presented the overall goal of the collaboration including the definition of the problem context for which the selection model is deemed valid, and then walked-through each step of the model. For each step, 1-2 high-level and open questions had been prepared to which the participants were invited to respond and, in general, to comment on the model, the steps, and each of the criteria. The focus group protocol is available in the Appendix (see Section Appendix) and the presentation material used is available on-line (Bjarnason 2021). The session was prepared by the first and the second author, who together led and moderated the session.

The focus group meeting took around 100 min though booked for 120 min to ensure sufficient time. Due to the ongoing Covid-19 pandemic, the focus group was held as a video conference (in Teams) where it was recorded. After the meeting, the audio was transcribed and analysed, and the main findings were discussed by the first and the second author and the model was updated accordingly resulting in the version described in Section 4.

About a year after the focus group and finalising the current version of the model, another research project within Ericsson used our model to select software. This application of the model was led by the second author and was performed independently from the other two authors. This example of use is described in Section 6, and provides an illustration of how our model can be of practical use.

## Software Selection Model

**Fig. 2:**
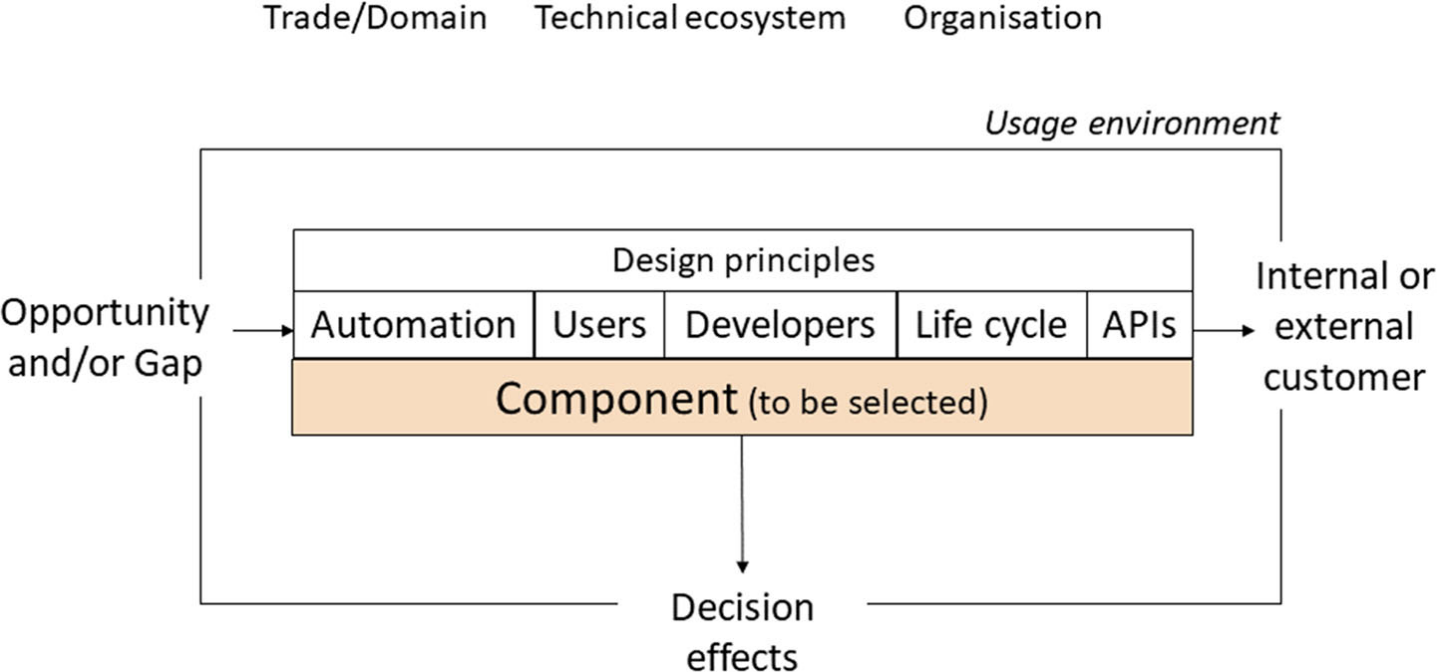
An overview of the problem domain for which our Software Selection Model was designed, and the context of component use for which the model has been designed

We have designed a model for selecting software components that is intended to provide decision support and guide practitioners in selecting software components by considering technical, business, and organisational aspects. The model is designed to aid and support organisations in procuring software components for their products and/or toolchains in a structured and evidence-based way. The model is applicable when a need, or an opportunity, arises to acquire new software components intended for use in an automated development set-up and/or used manually by human users, see Fig. 2. The need can be triggered either by an internal or external customer.

The model is designed to cover all the main aspects that affect a software component in use, such as technical and legal aspects. The usage environment of a component includes the context given by the business or trade domain, the culture and processes of the organisation, and existing technical ecosystems.

The usage environment poses requirements and constraints that need to be considered when selecting new components. Our model covers requirements both from the perspective of the developers that are to work with the components and those of the automated toolchains used during software development, e.g. for continuous integration. In particular, design principles for the following pose requirements on the software component under selection:

- The final automation solution involving the component and quality aspects of this.
- Human use of the component itself, e.g. through GUI or command line interface
- Developers that integrate the component
- Product life-cycle and the degree of support and alignment with the development life cycle for, e.g. design, implementation, configuration management, testing, project management.
- APIs, e.g. available functionality, supported form and format

The model was designed through an industry-academia collaboration where a scientific approach was applied to an industry-relevant problem. The model is the result of systematic analysis of existing research that was compared and combined with insights into industry practices. This combination of industrial and academic perspectives promotes relevance in research, and a systematic and scientific approach in industry.

### Overview of Process Steps and Roles

**Fig. 3:**
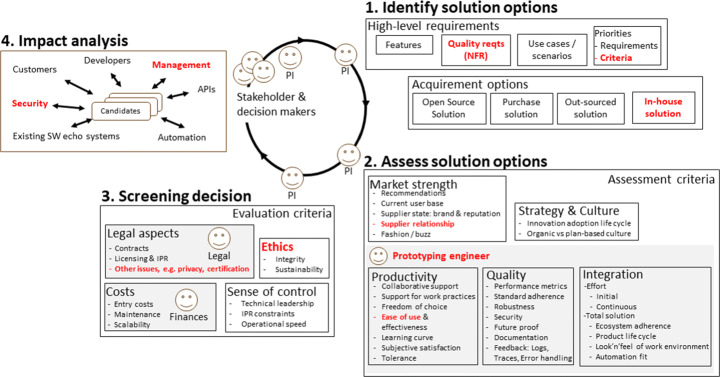
An overview of our software selection model consisting of a high-level four-step process and criteria for assessing and evaluating software components. Items in **bolded red** were added or modified based on feedback from focus group participants.PI - Principle Investigator

Our model consists of a four-step iterative process (roughly based on the selection process outlined in ISO/IEC 20741:2017) through which solution options are 1) identified, 2) assessed, 3) screened from a legal and business perspective, and 4) analysed for impact, see Fig. 3. For each step, the model contains recommendations regarding aspects to consider, in the shape of criteria for assessing and for evaluating solution options. In step 1, the high-level requirements and priorities for the component are identified and a set of possible solution options are identified. The following acquirement options are considered: open source, out-sourced or in-house development, or purchasing a solution. In step 2, information regarding the identified solution options is gathered guided by five categories of assessment criteria, namely market strength, strategy & culture, productivity, quality and integration. In step 3, the solution options are screened by assessing evaluation criteria and considering potential showstoppers from legal, financial, ethical or other strategic perspectives. Finally, in step 4 all relevant stakeholders are asked to perform an impact analysis of the solution options from their perspectives.

In each step, the gathered information is documented and provides input to decision makers. We recommend constructing and using a template for reporting the information gathered for each solution option including both qualitative and quantitative descriptions of the findings for each criteria (Phillips et al. 1998), i.e. both for the assessment criteria (used in Step 2) and for the evaluation criteria (used in Step 3). These reports aid decision making by providing an overview of the different solution options in a uniform format, and can also reduce future efforts. For example, if the selection process is repeated later on the information can be reused and relevant parts updated without redoing a full assessment (Church and Matthews 1995). For solution options that are rejected without a full assessment, the criteria that have not (yet) been assessed can be left blank in the report.

The four steps of the model are intended to be repeated until sufficient insight has been gained to make a decision regarding component selection. As new knowledge is gathered solution options can be removed at any step. If none of the original solutions are viable, additional ones can be added by iterating over the steps. In practice, we envision that at times some of the steps may be performed in parallel. For example, when acquiring a new software development tool at the team level, the assessment of solutions options (step 2) may be initiated already while identifying suitable options (step 1). In other cases, the assessment process may require more extensive investigations, in which case the steps are more distinct. An example of this is decisions on components to include in software products that affect a wider set of stakeholders, some of which may be company external and which often requires a more thorough investigation and a formal process.

Our model contains suggestions for roles and perspectives to include in different parts of the selection. We envision that a Principle Investigator (PI) leads and manages the work, and involves relevant stakeholders and decision makers as needed, e.g. in identifying the requirements for the solution (step 1) or in assessing certain aspects of a solution option (step 2). In step 2, we recommend involving a software engineer who is responsible for gathering insights into the solution options through prototyping and practical investigations. We call this role *Prototyping Engineer*. Through practical investigations into the solution options (Lester and Wilkie 2004), the main reasons for bad selection decision can be avoided, namely components failing to perform under stress in the targeted environment (Prather 1993). Also, we recommend letting senior practitioners who understand the intended use of the component and its context perform the evaluation through hands-on experience with the solution options (Mosley 1992). In the screening step (step 3), roles related to legal and finance need to be involved to ensure that the solution options are in-line with company strategies, e.g. regarding OSS licencing, and do not impose legal issues or unreasonable costs. We believe that this gradual filtering of solution options and gradually extending the number of involved stakeholders supports an efficient decision making process where the full set of stakeholders are not involved until in step 4. It is important to involve all relevant stakeholders in the final impact analysis step to ensure involvement and buy-in for the component-selection decision, but also to ensure that all angles and perspectives are considered.

### Step 1. Identify Solution Options

The selection of new software components is initiated by a gap and/or an opportunity, and thus a need for software components, e.g. to provide a new product feature or improving the efficiency of the development process. In this first step, the PI identifies feasible solution options based on the high-level requirements for the need and the requirements and constraints posed by the surrounding usage environment of the problem domain (Du Plessis 1993). The pros and cons of each available acquirement options should be considered and a set of potential solution options to pursue is defined as the output of this step. Thus, in this step the PI should:

1. Identify the need, opportunity or gap that is to be met by the new components, and the existing requirements posed by the problem domain, i.e. for the business, organisational and technical environment within which the component is to be used and relevant design principles that need to be adhered to.
2. Identify available and feasible acquirement options, i.e. open source solutions, purchase solution, out-sourced solution, in-house or in-house development.
3. Identify a set of solution options for the pursued acquirement options, based on the high-level requirements.

The information gathered in this first step may be obtained from a range of sources, such as literature searches, internet searches, experience reports and other material describing relevant components (Church and Matthews 1995; Delahaye and Du Bousquet 2015). The information may be publicly available or accessible for purchase, e.g. from the vendor or from independent evaluations of software components.

#### High-Level Requirements

The high-level requirements for the software component need to be identified to ensure that there exists a common understanding and vision of what is to be achieved by acquiring a new software solution (Prather 1993). We recommend identifying the high-level requirements through specifying what *features* and *use cases or scenarios* that are required, and the expected *quality* of and *priorities* for these requirements. While listing the desired features provides an overview of what functionality that is required, specifying user scenarios (or use cases) provides an effective way of capturing more detailed aspects of how the component is required to work in its context of use (Brown 1994; Le Blanc and Korn 1994) and can be evaluated through prototyping in step 2.

We suggest identifying the *priorities* of the assessment and evaluation criteria of our model according to the needs of the identified usage scenarios, and focusing the assessment on the most important criteria and thereby reducing the time and effort required to reach an informed decision. The context of intended use affects which of the many criteria that are the most important to consider in the current assessment. Identifying these *prioirities* allows focusing the evaluation efforts (Delahaye and Du Bousquet 2015). For example, for use in an educational setting the criteria *learning curve* and *tolerance* likely have a higher priority than for a component intended to be used as a tool within a software development team.

The requirements should cover:

- the features to be supported by the software component based on the identified needs, opportunities and/or gaps of the organisation (Church and Matthews 1995; Daneva and Terzieva 1996; Delahaye and Du Bousquet 2015), and
- the constraints posed by the problem context in the shape of the domain, technical ecosystem and organisation. This include design principles that must be adhered to.

While requirements should be at a sufficiently high-level to facilitate identifying suitable components, they should also be clear and specific enough to enable assessment of these in an objective fashion. Ideally requirements should be measurable, in particular regarding qualities such as “ease of use”, “portability”, “performance”. In addition, requirements should be defined in a uniform way to encourage objectivity and consistency in the assessment. Access to a common terminology can facilitate defining clear and unambiguous requirements (Brown 1994). For CASE tools, ISO/IEC 14102:2008 provides such standardised terminology. For software products and computer systems in general, the ISO/IEC 25010:2011 provides a consistent terminology for the quality aspects of software that can be used when specifying and assessing software quality.

#### Acquirement Options

A shortlist of solution options are identified by considering available suppliers and components that appear to match the high-level requirements, thereby performing an initial coarse evaluation that results in a set of components to be further assessed in later steps (Du Plessis 1993; Le Blanc and Korn 1994). Software components can be acquired from four main sources (Petersen et al. 2018) or a combination of these (Le Blanc and Korn 1994), either as

- an open source solution with available source code, commonly developed by a community,
- by purchasing the solution as a ready off-the-shelf commercial solutions (COTS), e.g. software product or service, usually without access to source code,
- through contract of outsourced development, or
- through in-house development, where the solution is developed within the organization.

The choice of acquirement option determines the amount of control an organisation has over the resulting solution, which can have a large impact on the development process and the organisations ability to evolve the resulting system for both COTS solutions (Torchiano and Morisio 2004) and for OSS solutions (Linåker et al. 2020). For this reason, other aspects beyond the necessary functional requirements need to be considered and the software ecosystem of the considered software components needs to be analysed w.r.t. stakeholder influence to understand the organisation’s ability to influence and interact with this ecosystem (Linåker et al. 2020). By performing a stakeholder mapping of the active stakeholders of the ecosystem and their interaction networks, their influence and agendas regarding the OSS component and subsequent directions regarding features and requirements in future versions of the component may be identified. Potential company strategies for use of and contribution to open source, regarding licensing policies (Krawatzeck et al. 2015), preferred suppliers etc. should be considered.

If no solution that matches the high-level requirements is found, extending and adapting an existing solution may be the way ahead. Ideally this is achieved by persuading the supplier to add or adapt new features to the existing solution thereby reducing the cost of maintaining the extension, or by performing in-house development if the organisation has the size and technical skill to do this is a cost-effective manner (Le Blanc and Korn 1994). For open-source software, there is the option to contribute the extension to the community and thus reduce the cost of maintaining the adaption.

### Step 2. Assess Solution Options

For each solution option, the PI leads an investigation of its strengths and weaknesses for the assessment criteria defined in our model. These criteria cover the aspects of market strength, productivity, quality, integration, and strategy & culture, see Table 3. The evaluation is performed through carefully reading available material (e.g. documentation, reviews, experience reports) and through prototyping the solution options (Delahaye and Du Bousquet 2015). The practical investigation is performed by a prototyping engineer who downloads and prototypes each solution option to assess the aspects of productivity, quality and integration for the use cases or scenarios defined in Step 1 (Delahaye and Du Bousquet 2015). In cases where evaluation copies of the component can not be obtained, a licence may be purchased to enable hands-on experience of the component, or the vendor may be invited to provide a demonstration (Church and Matthews 1995). Through prototyping, hands-on experience of the components under consideration can be gained and the practical utility of integrating and using the component can be assessed (Brown 1994). At this point of the assessment, detailed information regarding both strengths and weaknesses of the solutions is sought, and the solution options are ranked. The investigation should consider if and how requirements and criteria that are not met may be implemented, and at what cost (Le Blanc and Korn 1994).

**Table 3:** The assessment criteria (and supporting references) used in Step 2 to assess the solution options

Criteria	Sub-criteria
Market strength	
(Gezici et al. 2019;	∙ Recommendations
Adewumi et al. 2016)	(Le Blanc and Korn 1994; Kornecki and Zalewski 2005)
	∙ Current user base (Le Blanc and Korn 1994)
	∙ Supplier state (Alnafjan et al. 2013; Church and Matthews 1995;
	Du Plessis 1993; Le Blanc and Korn 1994)
	∙ *Supplier relationship (Taherdoost and Brard 2019)
	∙ Fashion, buzz (Suomi 1992)
Strategy & Culture	
(Le Blanc and Korn 1994)	∙ Innovation adoption
	∙ Planning vs organic culture (Prather 1993; Lester and Wilkie 2004)
Productivity (Du Plessis 1993)	
	∙ Collaborative support (Biffl et al. 2009; Rivas et al. 2008;
	Post and Kagan 2000; Gezici et al. 2019)
	∙ Support for work practices (Bashroush et al. 2017;
	Håkansson and Bjarnason 2020; Biffl et al. 2009; Rivas et al. 2008;
	Le Blanc and Korn 1994; Lester and Wilkie 2004)
	∙ Freedom of choice (Lending and Chervany 2002; Biffl et al. 2009;
	Lester and Wilkie 2004; Phillips et al. 1998)
	∙ *Ease of use & effectiveness (Bashroush et al. 2017;
	Håkansson and Bjarnason 2020; Petersen et al. 2018;
	Biffl et al. 2009; Church and Matthews 1995; Rivas et al. 2008;
	Pelechano et al. 2006; Lester and Wilkie 2004;
	Kornecki and Zalewski 2005; Post and Kagan 2000;
	Delahaye and Du Bousquet 2015; Phillips et al. 1998)
	∙ Learning curve (Håkansson and Bjarnason 2020;
	Torchiano and Morisio 2004; Church and Matthews 1995;
	Rivas et al., 2008; Le Blanc and Korn 1994; Phillips et al. 1998)
	∙ Tolerance (Du Plessis 1993)
	∙ Subjective user satisfaction (Håkansson and Bjarnason 2020;
	Church and Matthews 1995; Le Blanc and Korn 1994;
	Pelechano et al. 2006)
Quality	
	∙ Performance (Petersen et al. 2018; Le Blanc and Korn 1994;
	Prather 1993; Lester and Wilkie 2004; Kornecki and Zalewski 2005;
	Miller and Yeoh 2006; Gezici et al. 2019)
	∙ Standards adherence (Bashroush et al. 2017;
	Torchiano and Morisio 2004; Biffl et al. 2009; Brown 1994;
	Du Plessis 1993; Pelechano et al. 2006; Kornecki and Zalewski 2005)
	∙ Robustness (Du Plessis 1993; Kornecki and Zalewski 2005;
	Phillips et al. 1998)
	∙ Security (Petersen et al. 2018; Alnafjan et al. 2013; Du Plessis 1993;
	Krawatzeck et al. 2015; Post and Kagan 2000; Miller and Yeoh 2006;
	Gezici et al. 2019)
	∙ Future proof (Du Plessis 1993)
	∙ Documentation (Håkansson and Bjarnason 2020;
	Delahaye and Du Bousquet 2015; Du Plessis 1993;
	Le Blanc and Korn 1994; Pelechano et al. 2006;
	Lester and Wilkie 2004; Kornecki and Zalewski 2005)
	∙ Feedback (Du Plessis 1993; Miller and Yeoh 2006; Gezici et al. 2019)
Integration	
(Brown 1994; Du Plessis 1993;	∙ Effort, initial and continuous (Miller and Yeoh 2006)
Miller and Yeoh 2006)	∙ Ecosystem adherence (Håkansson and Bjarnason 2020; Brown 1994;
	Delahaye and Du Bousquet 2015; Le Blanc and Korn 1994;
	Kornecki and Zalewski 2005)
	∙ Product life cycle (Du Plessis 1993; Kornecki and Zalewski 2005)
	∙ Look’n’feel (Brown 1994; Du Plessis 1993; Rivas et al. 2008;
	Le Blanc and Korn 1994; Lester and Wilkie 2004)
	∙ Automation fit (Miller and Yeoh 2006; Gezici et al. 2019)

Criteria marked with * were added or modified after the focus group

For each criteria, information and assessment of how each solution option relates to these criteria are identified and documented. This information forms a qualitative assessment of the pros and cons of the solution options. If information is uncovered during this assessment step that indicates that the solution option is not viable, it may be removed from the set of solution options. The gathered information is input to the screening gate step and the (later) impact analysis.

The PI should optimise the selection process by considering the relative priority of the assessment criteria for the specific tool selection, and focus on assessing these criteria (Delahaye and Du Bousquet 2015). Similarly, Lundell and Lings pointed out that the set of tool characteristics in the CASE ISO standard is not complete or consistent for each particular evaluation, that human assessment and subjectivity is needed in the evaluation process, and argue for flexibility for organisations to adapt and customise evaluation frameworks to their specific needs (Lundell and Lings 2002). Similarly, Delahaye and du Bousquet in 2015 concluded based on a literature review that the comparison of software tools most commonly relies on the assessment of a combination of qualitative and quantitative criteria evaluated through applying tool candidates to a set of example cases (Delahaye and Du Bousquet 2015). We suggest that the evaluation process can be supported by standardised criteria, but that they need to be contextualised and prioritized for each specific organisation and case due to the large variations in organisational context and goals, and reasons for selecting a new software component. Furthermore, we pose that these criteria should be assessed through a combination of theoretical assessment (e.g. from information gathered from reports) and practical evaluation through prototyping of the solution options.

#### Criteria for Market Strength

Information regarding market strengths (and potential weaknesses) of the solution options include:

- **Recommendations** and previous experience of the solution from existing users could provide invaluable information, and something suppliers should be able to provide (Le Blanc and Korn 1994). Previous use of the component in similar contexts is especially valuable (Kornecki and Zalewski 2005).
- **Current user base**, e.g. size and degree of activity, and number of existing installations is an important indicator of the quality of the evaluated component (Le Blanc and Korn 1994).
- **Supplier state** and brand & reputation are important aspects to consider including the financial stability of the supplier since these factors indicate the supplier’s ability to provide good technical support and maintenance, e.g. through attracting and retaining competent personnel (Alnafjan et al. 2013; Church and Matthews 1995; Du Plessis 1993; Le Blanc and Korn 1994). Similarly, for OSS components, the stability and reputation of the community and the platform leader need to be considered. The long-term stability including maintainability of the component is affected by these aspects, e.g. if the component will be evolved and upgraded. However, the quality of the component does not always correspond to vendor size (Church and Matthews 1995) and assessors are advised to keep an open mind regarding components from smaller suppliers and to objectively assess the relevant criteria for components under consideration.
- **Supplier relationship** and previous experience of working with a specific supplier is an important factor to consider, which affects mutual trust and ease of communicating with the supplier, as well as, providing insight into their ability to deliver timely and high-quality software (Taherdoost and Brard 2019). If there is a pre-existing relationship that works well, selecting additional software from that supplier requires less investment and comes with lower risk than choosing to acquire software from a new unknown supplier.
- **Fashion & buzz** around a software component or tool, e.g. if the users or management have an inferior view of the components image, can affect its reception within the organisation, irrespective of the actual qualities of the component (Suomi 1992).

#### Criteria for Strategy and Culture

A component’s ability to align to the strategy and company culture of an organisation needs to be considered (Le Blanc and Korn 1994). In particular, the following aspects should be considered:

- **Innovation adoption life cycle** , i.e. the speed with which the component can be changed, replaced and released. Examples could be ecosystem adherence and open standards and interfaces.
- **Planning vs organic culture** relates to the level of discipline in an organisation and whether solutions tend to be designed and developed in a planned-based or in an organic fashion (as in agile). This is an important aspect to consider, in particular when selecting software tools which need to be assessed in-line with the specific culture and context in which they are to be deployed (Prather 1993). For example, a planned based organisation adhering to clear processes require either a tool that fully matches their process flow or that is flexible enough to allow their way of working. In contrast, an organisation where development is more organic may appreciate a tool that imposes a certain way of working and thereby implicitly stream lines, e.g. reporting between different teams. In organisations with cultural differences, e.g. sites with varying emphasis on the level of design versus coding, it is important to assess components ability to support and bridge these cultural differences.

#### Criteria for Productivity

The component’s impact on productivity needs to be assess from various perspectives including introduction and learning of the component, and the impact on productivity when the component is in use, e.g. for developers (Du Plessis 1993). This includes both factors for which the software provides direct support such as features for collaboration, and indirect support such as ease of learning and flexibility. Our model includes the following criteria related to productivity:

- **Collaborative support** and impact on collaboration, e.g. within the development organization, is an important factor, in particular for software tools and for distributed teams (Rivas et al. 2008; Post and Kagan 2000). This includes support for sharing data between users with adequate user and rights management facilities (Biffl et al. 2009).
- **Support for work practices** and how well the component supports existing work practices and development processes (Le Blanc and Korn 1994; Lester and Wilkie 2004), and supports a user in performing task in a comfortable way (Biffl et al. 2009) is an important criteria, especially for software tools (Rivas et al. 2008). When a software component is not aligned with current work practices this can increase the effort required to learn new technology and may also require changing the current work practices to adapt to a new software tool (Bashroush et al. 2017). Thus, work interplay is an important aspect of digital work environments that affects the effectiveness of the software engineers (Håkansson and Bjarnason 2020).
- **Freedom of choice** and the degree to which the component either enhances or limits the software engineers’ ability to make independent decisions (Phillips et al. 1998). The degree to which a software component restricts the software engineers work affects their subjective satisfaction, and can ultimately affect tool success (Lending and Chervany 2002). This factor also relates the configurability of a component and the ability to adapt the component to the user’s needs (Biffl et al. 2009; Lester and Wilkie 2004). The level of freedom of choice needs to be aligned with company strategy, and be balanced against other aspects such as security, quality assurance and process adherence.
- **Ease of use & effectiveness** concerns the usability of a software component (Petersen et al. 2018; Biffl et al. 2009; Church and Matthews 1995; Lester and Wilkie 2004; Kornecki and Zalewski 2005; Post and Kagan 2000) and its impact on the efficiency or performance of the component in use (Phillips et al. 1998; Church and Matthews 1995) and the overall cost of using these (Delahaye and Du Bousquet 2015). Usability needs to be considered with respect to the characteristics of the expected users which includes installing and tailoring, as well as, using a software component (Biffl et al. 2009). Understandability, e.g. of supported tasks (Rivas et al. 2008; Pelechano et al. 2006), complex data and information models, and is an important work environment factor (Håkansson and Bjarnason 2020) and a common limitation reported for CASE tools (Bashroush et al. 2017). This factor is particularly important for organisations with a high staff turnover (Rivas et al. 2008).
- **Learning curve** including good quality documentation and training material (Le Blanc and Korn 1994), and familiarity with the assessed software component need to be considered. Access to community-generated documentation (Glott et al. 2010; Samoladas et al. 2008), such as solution examples, is an important factor to consider since it affects ease of learning. The ease with which users can construct a mental model of the component (Phillips et al. 1998) affects productivity, either in using the component (Church and Matthews 1995; Rivas et al. 2008) or in integrating and extending it (Torchiano and Morisio 2004). Similarly, ease of learning is a work environment factor to consider for components that are to be used as part of a digital work environment (as tools) (Håkansson and Bjarnason 2020).
- **Tolerance** including fault tolerance, back-up and recovery features, the ease of managing system failures and issues (Du Plessis 1993) and how this affects the overall work productivity and effectiveness (above).
- **Subjective satisfaction** of the software engineers that are to work with and/or use the component is important to ensure that the solution can be used effectively, and is an important work environment factor that indirectly affects the organization’s productivity (Håkansson and Bjarnason 2020). The degree of user satisfaction can be obtained through prototyping, and developing full or partial demonstrations of the solution (Church and Matthews 1995; Le Blanc and Korn 1994; Pelechano et al. 2006).

#### Criteria for Quality

The quality of the component includes several aspects ranging from measurable quality properties of the component to qualitative assessments of the quality of the available documentation and feedback provided for the component. In particular, the following aspects should be considered, when relevant:

- **Performance** of the component can be assessed through metrics. Examples include response times (Le Blanc and Korn 1994), time for component to load and operate (Lester and Wilkie 2004), and the amount of resources required for a certain performance (Kornecki and Zalewski 2005). To correctly evaluate performance the component needs to be assessed in its targeted context and under realistic conditions regarding, e.g. number of users, amount of data, actual hardware (Prather 1993). Though this criteria is important, it if often overlooked in practice (as in (Lester and Wilkie 2004)) and is a common reason for selection decision failing and/or being perceived as negative (Petersen et al. 2018; Prather 1993).
- **Standards adherence** concerns the degree to which a component and its interfaces fulfill necessary standards and is important for the ability to integrate the component with other software (Torchiano and Morisio 2004; Brown 1994; Du Plessis 1993), exchange data between components (Biffl et al. 2009; Du Plessis 1993; Pelechano et al. 2006), and meet prescribed quality requirements, e.g. for process adherence (Du Plessis 1993) and certification for safety-critical software (Kornecki and Zalewski 2005). A solution option’s compliance with standards or quality policies and regulations within an organisation should thus be assessed (Bashroush et al. 2017).
- **Robustness** of the component is an important quality aspect that is especially important for embedded and safety critical systems (Kornecki and Zalewski 2005), but also from a user productivity perspective (Du Plessis 1993; Phillips et al. 1998). The criteria concerns the components ability to support the user in achieving goals including error prevention, recoverability, and provision of help (Phillips et al. 1998).
- **Security** of the system is another important criteria (Alnafjan et al. 2013; Du Plessis 1993; Krawatzeck et al. 2015) that sometimes is seen as less important (Post and Kagan 2000) and is often overlooked in practice. However, weak consideration of security may result in negative views of the selection decision (Petersen et al. 2018).
- **Future proof** and longevity of the solution and how easy it will be to maintain it, includes the component’s ability to adapt and expand to cover new standards, data formats, and methodologies (Du Plessis 1993).
- **Documentation** including the quality and amount of technical support and available information to aid integration and use of the component (Du Plessis 1993; Le Blanc and Korn 1994; Pelechano et al. 2006; Lester and Wilkie 2004; Kornecki and Zalewski 2005) can both affect the learning curve (Delahaye and Du Bousquet 2015) and work productivity, and ensures that important know-how is retained within an organisation (Håkansson and Bjarnason 2020). Availability and size of an active user community affects the amount and quality of documentation available, in particular example solutions and info on common issues (Glott et al. 2010; Samoladas et al. 2008). While assessing and comparing documentation quality can be difficult due to differences in format and style, it is important to review supplier documentation to verify its accuracy and adequacy (Le Blanc and Korn 1994).
- **Feedback** such as logs, traces, reports, and error handling (Du Plessis 1993), which affect the cost of integration and use of the component.

#### Criteria for Integration

This aspect covers the integration of the component in the existing software environment, both for the initial integration and the expected quality of this integration. This is a vital question to ensure that the selected component will be of practical use in the targeted (product or tool) environment, is portable and integrates well with other services, such as archiving and backup facilities (Brown 1994), and existing hardware environments and operating systems (Du Plessis 1993; Le Blanc and Korn 1994).

- **Effort** should cover the initial effort required to integrate the component, the continuous effort required to maintain the integration and the effort required to scale the solution.
- **Ecosystem adherence**, i.e. technical fit with existing systems and tools (Brown 1994; Du Plessis 1993; Le Blanc and Korn 1994) including compatibility (Delahaye and Du Bousquet 2015; Kornecki and Zalewski 2005) and system interplay (Håkansson and Bjarnason 2020) is an important factor that affects the cost of introducing and maintaining a solution option. Integration is facilitated when systems share a common philosophy of goals, design approaches, and implementation strategies (Brown 1994).
- **Product life cycle** relates to the component’s ability to support all relevant life cycle stages from design & development to maintenance and product end-of-life (Du Plessis 1993; Le Blanc and Korn 1994; Kornecki and Zalewski 2005). This relates to the criteria concerning support for work practices, but here the concern is on how well the component fits the overall life cycle of the development and project organisation.
- **Look’n’feel** of the work environment for manual interaction with the component(s) is important from the end-user perspective, and is a by-product of ecosystem adherence, but can also be supported by common design guidelines for user interfaces, data formats, error messages and customizability of the user interface (Brown 1994; Du Plessis 1993; Rivas et al. 2008; Le Blanc and Korn 1994; Lester and Wilkie 2004).
- **Automation fit**, i.e. how well the component(s) integrate in various automation toolchains.

### Step 3. Screening Decision

For each solution option, the value is assessed according to the evaluation criteria and a gating decision is made involving legal and financial stakeholders. Solutions that are not viable from the company perspective are removed. The remaining options are possible solution options.

In this step, the solution options suggested by the PI are screened by appropriate stakeholders w.r.t. legal and financial aspects, and solutions that are not viable from an organisational perspective are removed. The purpose of this step is also to align the component selection with overall company policy and to weed out unsuitable or unviable solutions from a company perspective. The screening decision is supported by a set of evaluation criteria provided by our model, see Table 4. While the assessment criteria primarily determine the technical and functional suitability of a solution option, the evaluation criteria capture harder parameters such as financial costs for procurement and usage, and the sense of overall control that the component yields. The outcome of this step is a set of viable solution options.

**Table 4:** The evaluation criteria (and supporting references) used in Step 3 to evaluate the viability of the solution options. Criteria marked with * were added after the focus group

Criteria	Sub-criteria
Legal aspects	
(Miller and Yeoh 2006)	∙ Contracts
	∙ Licensing and IPR (Krawatzeck et al. 2015)
	∙ *Other issues, e.g. privacy, certification (Kornecki and Zalewski 2005)
Costs	
	∙ Entry costs Du Plessis,^1993^; Petersen et al. 2018; Kornecki and Zalewski 2005;
	Church and Matthews 1995; Lester and Wilkie 2004; Brown 1994;
	Le Blanc and Korn 1994; Krawatzeck et al. 2015; Post and Kagan 2000)
	∙ Maintenance costs (Kornecki and Zalewski 2005; Brown 1994)
	∙ Scalability costs (Brown 1994; Church and Matthews 1995;
	Du Plessis 1993; Kornecki and Zalewski 2005)
*Ethical aspects	
	∙ Integrity (Maqbool and Herold 2021)
	∙ Sustainability (Adewumi et al. 2016; Becker et al. 2015)
Sense of control	
	∙ Technical leadership (Petersen et al. 2018; Torchiano and Morisio 2004;
	Linåker et al. 2018)
	∙ IPR constraints (Linåker et al. 2018)
	∙ Operational speed (Linåker et al. 2018)

#### Criteria for Legal Aspects

The legal aspects of a component option need to be considered, including **contracts**, **licensing and IPR**, and **other issues** of a legal nature. For example, privacy related issues. For safety-critical and other embedded software products, certification of components and tools used during the software development process is required and components ability and ease of providing this is an important aspect to consider in the evaluation process (Kornecki and Zalewski 2005). The exact requirements depend on the standards and regulations of the specific application domain but typically include guarantees for correctness and predictability of the components.

#### Criteria for Costs

The financial aspects of acquiring a specific component need to be considered since this is assumed to be a major financial investment, in particular for components which will be in used over an extended period of time by larger groups of users. The total cost for acquiring, integrating, and maintaining the component need to be assessed, i.e. the following:

- **Entry costs** include the initial cost of purchasing (Du Plessis 1993; Le Blanc and Korn 1994; Lester and Wilkie 2004; Post and Kagan 2000; Miller and Yeoh 2006) and/or adapting or developing the component (Petersen et al. 2018; Le Blanc and Korn 1994), costs related to integrating it in the existing environment (Brown 1994; Church and Matthews 1995; Miller and Yeoh 2006) and required training (Du Plessis 1993; Post and Kagan 2000). The cost of acquiring the component is among the most commonly used criteria in industry (Kornecki and Zalewski 2005). For open source components that can be acquired for free (Krawatzeck et al. 2015), it is important to also consider the other types of cost both for entry and for maintenance of the solution.
- **Cost of maintenance** including need of modifications (Kornecki and Zalewski 2005) to meet specific requirements, e.g. due to technology development of the component (Brown 1994) but also changes in the surrounding technical environment.
- **Scalability** of the solution also needs to be considered. For example, the component’s ability to scale to larger data sets and number of single and concurrent users (Du Plessis 1993; Church and Matthews 1995), and to manage evolving requirements due to changing needs and preferences for use (Brown 1994). In particular, components and tools developed in a research environment may not scale up well (Kornecki and Zalewski 2005), thus connecting this aspect to the criteria for market strength. Furthermore, one important factor is the technical impact on surrounding components and creating additional cost. A component with high impact typically provides less scalability.

#### Criteria for Ethics

The awareness of the importance of considering ethical aspects is increasing as software becomes omnipresent. This includes both the resulting application or system, as well as, the development of the software itself. Important aspects include personal **integrity** (related to user data) (Maqbool and Herold 2021) and **sustainability** of the overall software solution (Becker et al. 2015) in terms of environmental, social, and economic impact.

#### Criteria for Sense of Control

The degree of control that the organisation needs to and can have for a component option should be considered. This relates to a combination of business impact and control complexity, as pointed out by Linåker et al. In particular, the following aspects should be considered:

- **Technical leadership** and the level of innovativeness and novelty of the component, since this affects the number of available sourcing alternatives. A high degree of control is recommended, if the software component is unique and of high strategic and differentiating value, or imposes large negative effect if not available (Linåker et al. 2018). The ability to access the source code and control the evolution of the component should be considered (Petersen et al. 2018). For a purchased solution or an OSS component, this includes the ability to influence component evolution and feature priories (Torchiano and Morisio 2004).
- **IPR constraints** and other technology availability barriers. A lower degree of control can be accepted for commodity software and for components for which multiple alternatives are available, with low switching costs (Linåker et al. 2018).
- **Operational speed** relates to the component’s own life cycle and any life cycles that the component is in turn dependent on. For example, for maintenance releases that may be limited by time constraints in supplying the component but also by the organisation’s knowledge and capacity to absorb the technology (Linåker et al. 2018).

### Step 4. Impact Analysis

The main purpose of this step is to involve all relevant stakeholders within the organisation in an impact analysis of the solutions options to ensure that all perspectives and viewpoints are considered. In general, the decision processes for selecting new components should be aligned and harmonized within the overall organisation to avoid (later) conflicts and additional overhead and rework. An important part of this is managing expectations through continuous, factual and open communication and involvement of all stakeholders with a vested interest in the outcome, e.g. through publishing minutes, status reports, and allow adequate time for the upcoming changes to be understood and assimilated (Prather 1993).

Before making a final decision to select (or not) a specific software component, a wider set of stakeholders need be involved in performing an impact analysis of the solution options. The PI should supply the stakeholders with the information gathered for each solution option through-out the previous steps, i.e. high-level requirements, assessment and evaluation criteria. We suggest that stakeholders for the following aspects and perspectives are considered for involvement in this step, if relevant for the component selection at hand:

- Customers– internal and external
- Developers that will be integrating the component and/or using it in the development process
- Managers responsible for affected parts of the organisation
- APIs affected by the component
- Automation toolchain
- Existing software echo systems
- Quality metrics

The outcome of this impact analysis forms the basis for making a decision to either select a solution option, or, if no option is deemed good enough, to re-iterate the selection steps and identify and analysis additional solution options.

## Validation Results—Focus Group

The main purpose of the focus group was to validate our software selection model, but also to disseminate our research findings and provide value to the case company. Prior to the meeting, the participants received and reviewed a description of the model including the four steps and the assessment and evaluation criteria, and had thus prepared their feedback (one participant even sent us written feedback prior to the meeting).

At the meeting, the participants confirmed the overall approach of our model and the validity of the included assessment and evaluation criteria, while also providing industrial insights into the applicability of the model. Based on their feedback the model has been extended; by including prototyping as part of the assessment, by adding three assessment criteria and three evaluation criteria, and by making minor changes and extensions to steps 1 and 4. These changes are marked with red bolded text in Fig. 3. The findings and quotes (marked by *“italicised text”*) provided in this sub-section are based on statements made by the focus group participants.

### Overall: Problem Domain and Approach

The participants expressed that the problem addressed by the model is very relevant to their work *“both from the product side and the tool side”* and *“very valid for what we are doing”*. One participant expressed that they at least monthly have discussions related to software selection and another participant said that *“the selection of which tool [to use] maps to every single point in the picture [of the model].”* This participant also described that *“as developers and engineers... we often forget to make a broader analysis”* and that the model was of interest to consider in their work. An example given was being *“quick in taking on OSS utilities”* and then *“end up maintaining them”*, thus resulting in a higher cost long-term than was originally considered and ultimately having to *“live with the OSS restrictions”* and *“not having control”* of the long term cost and effort required for the solution. Another point that was highlighted was *“the need to consider legacy”* and how to handle *“existing interfaces. Do we need to maintain them, or ... [can we] phase some of them out?”*

While the participants thought the model provided a good way of working with these issues, they also pointed out that *“there is a big difference between products and tools.”* While the provided checklist was believed to provide good support *“if you want to deliver to a [customer] product... it might be different [for]... tools that are only used for development inside one company.”* This participants continued by describing that *“for products, the decisions are much harder and require more time, while tools are easier in some ways.”* However, as was pointed out by another participant, *“tools can also be delivered to customers... [making it] hard to distinguish between tools and components... it is all software!”* In conclusion, the focus group concluded that the model and the criteria are valid for both component and tool selection, though the model requires adapting to the specific context at hand.

### Modifications of Step 1 (Identify Solution Options)

The model was modified to highlight that *Priorities* in Step 1 refers to both priorities of the requirements and priorities of the criteria (of the model), and thus adapting the selection process itself. As was discussed at the focus group,, this would then support *scalability of the model* by enabling adapting the model to address the differences between selecting software for a product or for tool. This also allows to improving the cost-benefit ratio of a software selection, by allowing practitioners to *“select a few things from the checklist [the criteria]”* to focus on in the assessment.

Two other additions to step 1 were identified by the focus group participants, namely extending the model with an acquirement option for *In-house development* and adding *Quality (or non-functional) requirements* to the set of high-level requirements to identify in Step 1. As was pointed out by one participant, companies *“with a lot of money... [may consider] an in-house solution... [for which you] have full control”*, or a combination of an OSS solution that is modified through in-house development. *Quality requirements*, or non-functional requirements, are especially important to some domains, including that of Telecom within which our case company operates. The participants described lack of quality as an important motivator for software selection and one on which *“most of our decisions are based”*, and specially mentioned robustness, performance, stability, and usability as important quality aspects.

Furthermore, based on feedback from the participants the order of the two activities in step 1 have been changed to indicate that the high-level requirements should first be clarified before identifying potential solution options. This also highlights that *“the initial requirements and constraints will reduce the amount of options”* that are selected for further assessment, thereby focusing the effort only on the options that meet the high-level requirements.

### Modifications of Step 2 (Assess Solutions Options)

A major point that was brought up at the focus group was the importance and role of prototyping when selecting software, which led to adding this aspect to Step 2. As was described by a participant, no matter if the aim is to acquire a solution through in-house development or other sources, *“we always prototype”* as a way to gain competence and insight into what is needed. The role of a prototyping engineer has been added to the model in response to this feedback. This role is responsible for the assessment of the technical criteria of *Productivity*, *Quality*, and *Integration*. Through prototyping, information regarding *“how much effort it is to integrate the software”* can be obtained, as well as, learning whether or not the software *“is fast and stable enough”*, i.e. of sufficient quality. In addition, insight into ease of use and look’n’feel are gained through *“working with the tool at a more practical level”* including the quality of the documentation. The two remaining categories of assessment criteria, i.e. *Market Strength* and *Strategy & Culture*, were agreed to *“be the ones that can be assessed without prototyping”*. However, this participant also emphasised that these criteria *“have a very important place in the selection.”*

### Assessment criteria of Step 2 (Assess Solution Options)

The proposed assessment criteria were seen as *“as a very good check list of things you need to consider”* and thus were validated by the focus group participants. In addition, two additional criteria were brought up which have now been included in our model, namely:

- Productivity: Effectiveness and Ease of use
- Market strength: Supplier relationship

One participant pointed out that usability, or ease of use, was missing in our model. Based on this, we decided to explicitly include *ease of use* as part of *Effectiveness*, even though this aspect was already implicitly included in other usability-related criteria, such as *Freedom of choice*, *Learning curve*, and *Subjective satisfaction*. In this way, this important perspective is more clearly visible to practitioners using our model.

One participant pointed out that inter-company relationships and previous *supplier relationships* are an important aspect to consider as part of *Market strength*. Collaborating with an existing supplier *“feels a lot safer”* than *“selecting a new supplier that we have never worked with.”*

### Evaluation criteria of Step 3 (Screening Decision)

Our focus group participants agreed to the evaluation criteria of our model, and brought up three additional aspects that we have now included, namely

- Other legal aspects such as privacy
- Ethics: integrity and sustainability

One participant pointed to ethics as an important category of criteria to include to avoid *“buying software from a company that is behaving in an unethical way.”* This aspect relates to a company’s ethical policy and the image they want to convey, e.g. as a sustainable company, or being concerned with personal integrity.

In addition, the category containing financial criteria was renamed *Costs* to better communicate its content.

### Modifications of Step 4 (Impact Analysis)

Only minor changes were identified for Step 4, namely to add *Security* as a stakeholder and to rename *Line manager* to *Management* for the model to be more generically applicable to different organisational set-ups. As was pointed out by one participant, *“there are many constellations”* of management, and in some companies product management are those making software selection decisions rather than line managers. Adding stakeholders for security aspects, is due to the increased awareness of the *“the importance of these [aspects]... [to ensure that] the end product will not be broken or hacked by anyone*”.

## Validation Results—Example of Use

We here provide an example of using our model to select software at Ericsson based on a need to extend the capabilities of an existing engineering tool (Tool A) within one of the company’s research projects. Tool A, as well as the planned extension of it, is to be used directly by developers and within automated development toolchains within the company. The new software does not necessarily have to be self-contained, but may consist of sub components. The specific names of Tool A and the assessed software components have been anonymised for confidentiality reasons.

The main stakeholders of this selection are the tool developers of the company (responsible for maintaining Tool A and other software tools) and the researchers of the project selecting the software. The PI of the research project (the second author) performed the selection process together with a software engineer from the company assigned the role of prototype engineer.

The problem domain (see Fig. 2) consisted of the software development tool environments at Ericsson, including industry standard design principles for automation and APIs, and common work practices for the software development life cycle.

The software selection was guided by our model and performed in the prescribed four steps, as described herein together with the results and experience of using the model. In this example, the selection was performed in two iterations. In the first iteration, three solution options were investigated but since they did not meet the high-level requirements the investigation did not proceed to step 2. Instead, the selection process was repeated. In the second iteration, the requirements were adjusted to a sub-set, and some additions were made, to enable searching for a good fit for a tool that could be extended through in-house development. After performing step 1-4, this second iteration resulted in selecting a combination of open source software and in-house development to meet the need for extended tool functionality.

### Step 1: Identify Solution Options

The feature request originated from an internal customer, and the technical environment and design principles used in this selection were derived from the existing Tool A. Since Tool A is open source software (OSS), identifying an open source solution was an important requirement. The technical requirements consisted of the ability to parse a standard format and transform it to an intermediate format that can accommodate the needs of (other) in-house developed components. The criteria proposed by our selection model were considered and the most prioritised criteria were used in the subsequent steps 2 and 3.

The acquirement options of “Open Source” and “in-house that can be open sourced” were selected in the first iteration. The prototype engineer investigated various solutions and tested a few OSS components based on the criteria Productivity, Quality and Integration. Three viable solution options were identified, namely two open source components, S1 and S2, and one in-house solution, S3. However, these solution options only fulfilled parts of the requirements. Thus, in the second iteration, the requirements were revised to fit this situation, and the acquirement option of a combination of open source software and in-house development was sought. This resulted in identifying (the same) S1-S3 as solutions options.

### Step 2: Assess Solution Options

In this step, the solution options S1, S2 and S3, were assessed according to the prioritised criteria, see Table 6. The assessment was based on exploring the solutions through prototyping, and on available information and knowledge of the components.

### Step 3: Screening Decision

For this step, the evaluation criteria for legal, ethical, cost, and sense of control were used to evaluate S1–S3. There was only one solution option that met all prioritised criteria, namely S1, according to the following:

- **Legal** Licencing and IPR: Since S1 is already in use within Tool A, the open source licenses are compatible with current strategy.
- **Ethics** Since S1 is already approved for use within the company, sustainability, integrity and security aspects were deemed okay.
- **Cost** Entry cost was deemed acceptable though an initial integration development effort is needed. The maintenance costs are predicted to be low since the APIs and formats of this solution option have a history of being stable. From a scalability standpoint, the extended tool can be seen as internal within Tool A and therefore does not affect scalability.
- **Sense of control**
  - **Technical leadership** Regarded as “good enough” since Tool A is neither part of the company’s core business nor is the solution unique.
  - **IPR constraint** Tool A has already been subjected to an IPR evaluation. Extending this tool further was not deemed affect that evaluation.
  - **Operational speed** was deemed as being a good fit since the release cycle of the OSS project for C1 adheres to that of Tool A.

### Step 4: Impact Analysis

Extending Tool A “in-house” using the open-source component S1 fulfilled all the prioritised criteria and a decision was made by the PM of the research project (also the PI of the selection process) to pursue this solution option. The majority of the impact analysis became implicit already in step 2, since the extended tool will be part of Tool A. However, due to the extended set of features the users of Tool A were consulted. The new features of Tool A can be accomplished by extending rather than modifying existing APIs. For this reasons, adopting the solution option was deemed to be “more of the same” and require only minor updates to the current source code. Therefore, since the impact for the stakeholders and decision makers was similar to that of tool A, this did not require any further analysis.

### Practitioners Experience of Using the Model

The practitioners described that at first glance, the selection model appears to be simply common sense and with a good fit to their internal processes. As they started to use the model, and the criteria therein, they discovered that the model provides a very good, easy-to-use, and condensed check list for selecting software. The model triggered relevant discussions and valuable activities in analysing needs and strengths and weakness of the assessed solution option. The practitioners also found that the model helped them to keep their discussions objective and fact-based, and focused on the most relevant aspects of the software solution and of their needs. While the practitioners selected a prioritised sub-set of the provided assessment and evaluation criteria (as prescribed by the model), they found no need to consider additional aspects or criteria. Thus, in this case the model provided sufficient support.

## Threats to Validity

There are several risks related to the validity of our research (as for any study), in particular related to *researcher bias*, and to the *generalisability*, *completeness*, and *relevance* of the presented software selection model. The risks related to *biases* concern possible misunderstandings of the collected data, e.g. what the practitioners actually meant at the focus group and in interpreting the articles used as input to our design. The risk of misunderstandings at the focus group was reduced by preparing an overview description of our model that was sent out prior to the focus group meeting, thereby providing the participants with the opportunity to prepare and increase their ability to ask clarifying questions at the focus group. The data gathered during the focus group was analysed by the two first authors, first by jointly identifying the main outcome of the focus group and the subsequent changes to the design, and later through transcribing the meeting and coding this transcription. Furthermore, the risk of misunderstandings, at the focus group and throughout the study, was mitigated through frequent meetings and discussions through which a common terminology and understanding of each other’s viewpoints was established. This close collaboration, thus led to an alignment between the academic and the industry participants, which partly mitigated the risks to internal validity. Furthermore, including the third author in the final design iterations provided a triangulation of our design against this author’s understanding of the domain and through further rapid reviews.

The *generalisability* of our software selection model and the extent to which it may be valid and applicable to other companies (besides the case company) has not been validated, and remains as future work. The applicability to other companies, e.g. of similar size and type of software development, needs to be assessed on a case by case basis. Since the case company covers a wide range of different types of projects and software development methodologies, we believe that it is very likely that our model is applicable to a wide range companies of similar sizes and similar products domains as our case company. Furthermore, the fact that our work is based on previous research from a wide range of domains and case organisations also indicates that our software selection model may be applicable beyond the scope of our one case company. The model was designed for the wide range of software products and varying types of development processes found within Ericsson. We briefly describe some of the important dimensions of the case company (see Table 5), that may help a reader to judge the likely applicability of this approach to their own context.

**Table 5:** Some details of the case company

Dimension	Details
Domain	Telecommunication
Application type	Several types from real time systems to pure software
	applications and tools
Component source options	Various, including: inhouse, outsourcing, commercial
	off the shelf components, open source software
Processes	Various including CI/CD pipelines, and Agile and
	iterative development mixed with quality gates
Standards and Certifications	Various
Size of the company	Very large
Life cycle stages	All, including new development, maintenance, legacy system

The *completeness* of the presented software selection model has not been fully evaluated, and there may be additional factors and criteria beyond the ones that we have identified through this work. Some additional factors were mentioned during the focus group. Some of these have been added to our model, e.g. quality requirements and prototyping. Some of the mentioned aspects, such as ease of use, were already represented in the model by being included in other criteria such as effectiveness (part of productivity). No additional criteria were needed in the practical application of our model at the case company. However, further validation of the model through applying it to other cases and in other contexts is required to test and possibly improve its completeness.

We judge the *relevance* of our research as high due to involving practitioners throughout our study, both in defining and preparing the research, and in designing and validating the resulting selection model. The second author works at Ericsson and has contributed to this study with deep insight and knowledge of large-scale industrial software engineering based on his long work experience. He has also implicitly and explicitly involved others at the case company in the study, both through internal meetings and discussions at Ericsson related to this study, and through facilitating a focus group with representatives from one of Ericsson’s teams responsible for software engineering tools. There is a threat that the validation results of the focus group may be biased as the practitioners may consider criticising the model impolite. We did not observe any signs of this as practitioners discussed several aspects of the model and, as reported in Table 3, and even suggested additional criteria. While we provide a practical example of use of our model, further research is needed to fully evaluate the applicability of the model in practice.

## Conclusions

Software developing organisations frequently make decisions regarding which software to acquire and use in their products and in their software development environments, e.g. in the toolchains enabling continuous software engineering. However, research shows that in industry today these decisions tend to be made in an ad hoc manner, and existing research on, e.g. component engineering and on tool selection, has a weak uptake by practitioners. Therefore, we have investigated this topic through a joint research study and through a close collaboration between industry and academia.

Our research study used a method engineering approach, where current knowledge was queried through rapid reviews and used as input to the iterative design of a new model for selecting software. Relevance of the research study and of the designed model was ensured by active participation from industry for all stages of our study, from preparations through design to validation of our model. In particular, the second author (employed by the case company) has participated throughout the study. In the final stage of the study, the model was evaluated through a focus group with three additional practitioners from the case company, and was later used within the company (independently of the researchers) to assess and select software solution for an engineering tool.

Our software selection model is designed to provide decision support and guidance in selecting software by considering, not only technical, but also business and organisational aspects. Thereby, our model provides support in assessing software from a holistic perspective. The model consists of a straight-forward four-stage process from identifying solution options to a full impact analysis of these, and criteria for assessing and for evaluating these solution options. In total, our model contains nine categories of criteria, namely assessment criteria for market strength, strategy & culture, productivity, quality, and integration, and evaluation criteria for costs, legal aspects, ethical aspects, and sense of control.These criteria and the process elements upon which our selection process is based, draw on the knowledge and validity of previous research, while providing a novel contribution in combining existing knowledge in the co-design of our proposed software selection model.

Initial validation of the model through a focus group and by applying the model at the case company confirm the practical relevance of the model for selecting software components, and that practitioners can use the model to select software for use either in business products or in software engineering tools. While production software and software tools share some criteria, e.g. costs, the priority and interpretation of other criteria may vary, e.g. for productivity. Our model is designed to be applicable to all types of software use through adapting and prioritising the set of main criteria used to assess and evaluate the solution options. For this reason, prioritisation of both requirements and criteria are prescribed for the initial step of the model, where the high-level requirements and suitable acquirement options are identified.

Future work includes evaluating and applying the model to additional cases and contexts, thereby strengthening its validity and further improve its design. This will also provide an opportunity to gather further empirical data on decision making around software selection in industry. Another interesting topics is to investigate if and how the importance and prioritisation of different criteria vary depending on contextual factors, such as target of the selected software (product or tool) but also depending on product domain, size of company etc. Furthermore, additional research on individual criteria, how to assess them and their impact on business and development processes provides interesting avenues for future research.

Finally, our study demonstrates a way of performing industry-relevant research with a high degree of engagement from industry. Throughout this study, industry and academic partners worked “as one team”; from defining study goals and research approach, through data collection and design to analysis and reporting of the results. This close and mutual collaboration was supported by an iterative approach that enabled us to gradually design a solution (our model) to a problem experienced by industry. Furthermore, the use of Interactive Rapid Reviews provided a method for injecting scientific knowledge into the design process. Through these reviews, the researchers used their competence to identify relevant research articles and present these to our industry partner, thereby making previous research more easily accessible. A solution was then derived through a mutual design process that was supported by scientific literature, which was compared to and complemented by industry experience. Our focus participants confirmed the relevance and usefulness of the resulting model that they believe provides good support for their selection decisions. This response also validates that our collaborative research approach is inductive to relevant research results.

## Data Availability

The datasets for the rapid reviews and the presentation material used in the study (intermediate results of rapid reviews and focus group) are available on-line (Bjarnason 2021). Data collected during the focus group are not publicly available due to reasons of confidentiality.

## Appendix A: Focus Group Protocol

**Introduction—5 min**

- Welcome! Context of ongoing collaboration and specific study on software component selection.
- Today’s agenda: goals and purposes
- Research participation, recording etc.
- Who we are including participants

**Model presentation incl questions for clarification—15 min**

- Purpose: to provide support and guidelines in preparing for selection decision. Not a strict process, rather free to adapt to specific needs.
- The steps
- The roles: PI, stakeholders (e.g. tooling department, architects, sw devs), decision makers (line managers, legal and financial experts or departments, technical experts)

**Problem domain—10 min**

- Question: How does this problem domain relate to your work?

**Model, overall and work flow—15 min**

- Question: Does this model make sense to you, the flow?

**Model, criteria—15 min**

- Question: Do the criteria in this model make sense to you?

**Main focus—10 min**

- Question: What in this model do you want to focus on more in your work?

**Improvement focus—10 min**

- Question: What can be improved in this model?

## Appendix B: Assessment of Criteria for Example Case

**Table 6:** Evaluation of assessment criteria for example case

Criteria	S1	S2	S3
Market strength			
Current user base	Very large	Medium/small	None
Supplier state	Stable	Unclear	Very unstable
Fashion / buzz	Very talked-out, good reviews	Somewhat talked about	N.A.
Strategy and culture			
Organic vs plan-based	Mix	Organic	Wanted: organic
Productivity			
Collaborative support	More complex than S3. Requires	Compared to S1, less complex.	Similar to S2, though lower
	intermediate format.		requirement due to in-house solution.
Support for work practices	Prototyping revealed that there are no major differences w.r.t. to criteria between the solution options.		
Quality			
Standard adh., robustness	High for parsing functionality.	Less robust and less adherant to	TBD
	Almost a standard due to size of user	standards. Use and nature of needed	
	community.	format reduces the need for this criteria.	
Future proof	Very good due to very stable OSS project.	Hard to judge	Not future proof, unless handled
			internally
Documentation	Very well documented	Quite well documented	Requires documentation effort
Integration			
Effort: Initial	High (intermediate format issues cause	In between S1 and S3	Low (no need for intermediate format)
	substantial work)		
Effort: Continuous	Low (intermediate format assumed	In between S1 and S3	High (keep up with standard formats)
	stable		
Echo system adherance	Good due to common OSS component	Weak due to subcomponents not	Requirement on the in-house solution
		aligned with existing ecosystem	
Automation fit	Good	Good though risks due to more	Requirement on the in-house solution
		external dependencies than S1	
